# Understanding Willow Transcriptional Response in the Context of Oil Sands Tailings Reclamation

**DOI:** 10.3389/fpls.2022.857535

**Published:** 2022-04-27

**Authors:** Abdul Samad, Gervais Pelletier, Armand Séguin, Dani Degenhardt, Douglas G. Muench, Christine Martineau

**Affiliations:** ^1^Natural Resources Canada, Canadian Forest Service, Laurentian Forestry Centre, Québec City, QC, Canada; ^2^Natural Resources Canada, Canadian Forest Service, Northern Forestry Centre, Edmonton, AB, Canada; ^3^Department of Biological Sciences, University of Calgary, Calgary, AB, Canada

**Keywords:** oil sands tailings, reclamation, transcriptome, willow, polycyclic aromatic hydrocarbon

## Abstract

One of the reclamation objectives for treated oil sands tailings (OST) is to establish boreal forest communities that can integrate with the surrounding area. Hence, selection of appropriate soil reclamation cover designs and plant species for revegetation are important aspects of tailings landform reclamation and closure. Research and monitoring of the long term and immediate impacts of capped OST on the growth and survival of native boreal plant species are currently underway. However, plant responses to OST-associated toxicity are not well known at the molecular level. Using RNA sequencing, we examined the effects of three types of OST on the willow transcriptome under different capping strategies. The transcriptomic data showed that some genes respond universally and others in a specific manner to different types of OST. Among the dominant and shared upregulated genes, we found some encoding protein detoxification (PD), Cytochrome P450 (CYPs), glutathione S-transferase regulatory process (GST), UDP-glycosyltransferase (UGT), and ABC transporter and regulatory process associated proteins. Moreover, genes encoding several stress-responsive transcription factors (bZIP, BHLH, ERF, MYB, NAC, WRKY) were upregulated with OST-exposure, while high numbers of transcripts related to photosynthetic activity and chloroplast structure and function were downregulated. Overall, the expression of 40 genes was found consistent across all tailings types and capping strategies. The qPCR analysis of a subset of these shared genes suggested that they could reliably distinguish plants exposed to different OST associated stress. Our results indicated that it is possible to develop OST stress exposure biosensors merely based on changes in the level of expression of a relatively small set of genes. The outcomes of this study will further guide optimization of OST capping and revegetation technology by using knowledge based plant stress adaptation strategies.

## Introduction

Hydrocarbon extraction from the oil sands generates large volumes of oil sands tailings (OST) in the Athabasca region of northern Alberta, Canada (Azam and Rima, 2014). The OST contains several constituents of toxicological and ecological concern (CTEC) including naphthenic acids, trace metals, salts and residual bitumen that can negatively impact the vegetation community (Timoney and Ronconi, 2010; Wang et al., 2014; Parrott et al., 2019). Development of soil reclamation cover (capping) designs and revegetation strategies adapted to the OST substrate is important for OST landform reclamation and closure. With progressive reclamation of OST occurring, the oil sands industry must adaptively manage, develop and refine best management practices for the reclamation of OST (Badiozamani and Askari-Nasab, 2014).

Fluid tailings, a by-product of bitumen extraction, are composed of a mixture of water, sand, fine silts and clays, salt, residual bitumen and dissolved organics (Kasperski, 1992; Chalaturnyk et al., 2002). The terrestrial reclamation of fluid tailings can only begin after the tailings are dewatered and consolidated. After sufficient consolidation, the deposit will be capped with sands and/or overburden prior to any reclamation activities (Badiozamani and Askari-Nasab, 2014). Several technologies have been developed to facilitate the consolidation of fluid tailings including centrifugation, in-line thickening, mixing fluid tailings with Clearwater Formation saline-sodic shale overburden, and natural processes such as transpiration by plants (BGC Engineering Inc, 2010; Wang et al., 2014). During centrifugation, fluid tailings are pre-treated with gypsum and/or polymer to form larger flocs that settle quickly in the centrifuge and separate from the water. This process creates centrifuge tailings (CF) that consist of >55% solids. Adding Clearwater Formation overburden to fluid tailings generates a consolidated tailings called co-mix (CM) tailings. The process results in the absorption of pore water from fluid tailings into the high clay and silt content of the overburden material to create a substrate with solids content between 68 and 75%. Another process to facilitate consolidation is adding a thickener through secondary inline chemical treatment to the middlings (a coarser tailings) prior to deposition, thereby creating a thickened tailings (TT) with sand to fine ratio (SFR) of 0.5–2 and a solids content >65% (BGC Engineering Inc, 2010; Wang et al., 2014). These various types of treated fluid tailings have distinct chemical properties and may pose unique challenges and opportunities for reclamation.

Even after partial dewatering treatments, tailings may not be suitable for plant growth due to CTEC-induced phytotoxicity, high salinity and low nutrients (Wolter and Naeth, 2014; Lalonde et al., 2020). The placement of a soil reclamation cap [e.g., native surface soils such comprised of mineral substrate (till) and/or peat mineral mix (PMM)] over treated tailings may improve plant establishment by reducing the level of OST-associated abiotic stresses (Hargreaves et al., 2012; Wolter and Naeth, 2014; Huang et al., 2015). Many studies reported the positive impact of soil reclamation capping materials on plant growth and development (Hargreaves et al., 2012; Wolter and Naeth, 2014; Huang et al., 2015; Lalonde et al., 2020). A soil reclamation cap serves as suitable medium for root development which can support plant growth in several ways, such as providing organic matter and essential plant nutrients and improving water retention and aeration (Lalonde et al., 2020).

The selection of appropriate plant species is vital for rapid revegetation and reclamation of industrially disturbed sites. Willows (genus *Salix*) are becoming increasingly important for this purpose due to their potential for phytoremediation and ecosystem restoration (Kuzovkina and Quigley, 2005) and high tolerance against various environmental stresses (Kuzovkina and Volk, 2009; Mosseler et al., 2014). Beaked willow (*Salix bebbiana* Sarg., 2*n* = 38) is native to Canadian landscapes, widely distributed across Canada, and well adapted to boreal climates (Argus, 2007). It is very common throughout Alberta where it colonizes various types of ecosystems (Limited, 1989). *S. bebbiana* is well adapted to a range of moisture conditions, from well drained to waterlogged soils, and is commonly found as an early colonizer after a disturbance (Limited, 1989). Recently, *S. bebbiana* has been reported as a potentially suitable species for OST reclamation and showed high survival rate in un-capped tailings but significantly different growth responses in capped and un-capped treated tailings (Lalonde et al., 2020). Although, various visual plant stress symptoms, including chlorosis, necrosis and stunted growth of willows, were observed in different types of OST (Wolter and Naeth, 2014; Lalonde et al., 2020; Li et al., 2020), the molecular-level responses of willows to multiple OST-associated stresses are yet unclear. Recently, RNA sequencing (RNAseq) technology has emerged as a powerful tool that facilitates the identification of expression patterns and regulatory mechanisms of differentially expressed genes (DEGs) in plants (Xia et al., 2021).

Here, we aimed to understand the transcript level responses of willow (*S. bebbiana*) grown on various types of consolidated tailings (CF, CM, TT) with or without capping of various thicknesses and designs to identify common genes and key regulatory pathways linked to multiple types of stresses. This integrated study provides insights into the common molecular mechanisms of tailings-induced stress responses in willow, which can be used to develop simple and cost-effective monitoring tools for stress detection in plants grown in tailings. Rapid detection of tailings related phytotoxicity could help to alleviate plant stress by modifying capping treatments and implementing plant stress adaptation strategies.

## Materials and Methods

### Tailings and Capping Materials

Three types of tailings (CF, CM, and TT) and two types of reclamation capping material [peat mineral mix (PMM) and till (mineral substrate)] were evaluated in this study. Selection of capping material used for each type of tailings was done in consultation with the oil sands companies based on suitability and on-site material availability. The basic physicochemical properties of tailings and reclamation capping materials are summarized in Supplementary Tables 1, 2, respectively. For tailings, concentrations of plant available nutrients, trace metals, and petroleum hydrocarbons (PHCs) [including naphthenic acids (NAs) and bitumen contents] are also provided in Supplementary Table 1.

**FIGURE 1:**
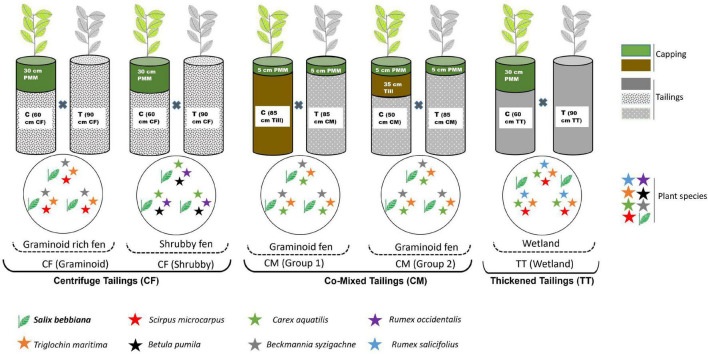
Diagram of experimental design in which willow (*Salix bebbiana*) was grown in different types of oil sand tailings (with or without capping of different depths) along with various plant communities. Gray plants represent the signs of OST-related stress (i.e., columns covered with no reclamation cap or 5 cm of reclamation cap) and green plants represent no sign of stress (i.e., columns with ≥30 cm of reclamation cap). Four columns were assembled for each treatment (*n* = 4). T, treatment; C, control; CF, centrifuge tailings; CM, co-mix tailings; TT, thickened tailings; PMM, peat mineral mix; Till, mineral substrate (classified as sandy clay loam soil with low organic carbon).

To study transcriptional responses of willow to OST, capping treatments in which willows showed signs of OST-related stress (i.e., columns covered with no reclamation cap or 5 cm of reclamation cap) were compared to controls in which willows showed no sign of stress (i.e., columns with ≥30 cm of reclamation cap). A complete description of the capping treatments tested for each type of tailings is provided in Figure 1. These capping treatments were selected for comparison purposes and take into account the limited size of the columns. They do not represent current or expected on-site reclamation capping requirements for these tailings.

Centrifuge tailings and CM tailings and capping materials (PMM and till) were received from Syncrude Canada in October, 2018. Columns (227 L, 90.5 cm × 51.4 cm) with various thicknesses of these two types of tailings and capping materials (Figure 1) were assembled in late October, 2018 at CanmetENERGY (Devon, AB, Canada). TT and capping material (PMM) were received from Imperial Oil Ltd., and used to assemble the TT columns (Figure 1) in early May, 2019. A total of four replicate columns were prepared for each treatment.

### Plant Growth Conditions and Sample Collection

Willows (*S. bebbiana*, target species for this study) cuttings were planted in columns of all types of tailings (CF, CM, TT) accompanied by other boreal plant species (Figure 1) between April and May, 2019. The cuttings were collected near a tailings pond the season prior to the start of the study, propagated to establish roots (∼6 months), and kept dormant until it was ready to be planted. The columns were placed in two greenhouse bays of the Northern Forestry Centre (Edmonton, AB, Canada) having comparable temperatures (Supplementary Table 3), with a photoperiod of 12 h. Supplementary lighting was used during time periods of non-optimal solar radiations. The height of willows was documented just before sampling at the end of 1st growing season (September, 2019) and showed a clear growth difference between capping treatments (Supplementary Figure 1). Plant leaves for the study of tailings stress-responsive gene expression in willow were collected by clipping multiple fully developed leaves from a single plant (*n* = 4, from each column). The leaves were placed in 50 ml Falcon tubes, snap frozen in a dry ice-ethanol bath, shipped to the Laurentian Forestry Centre laboratory (Québec City, QC, Canada) on dry ice, and stored at −80°C.

### RNA Extraction and Sequencing

Leaf samples of willow were ground and homogenized in liquid nitrogen with mortar and pestle. Total RNA was extracted from 100 mg of ground tissue using the RNeasy Plant Mini kit (QIAGEN, Toronto, ON, Canada, Cat. #74903) with in-column RNase-Free DNase Set (QIAGEN, Cat. #79254) according to manufacturer’s instructions, with the exception of a modification to the lysis buffer (RLT, QIAGEN, Cat. #79216). The lysis buffer was prepared as described (MacKenzie et al., 1997) to improve RNA yield. The DNA-free RNA extracts were quantified using the Qubit 4 fluorometer (Thermo Fisher Scientific, Waltham, MA, United States) with the Qubit RNA BR Assay Kit (Thermo Fisher Scientific, Cat. #Q10211). The RNA integrity number (RIN) was tested with the Agilent RNA 6000 Nano Kit (Agilent, Santa Clara, CA, United States, Cat. #5067-1511) on a 2100 Bioanalyzer instrument (Agilent, Cat. #G2939BA). All RNA samples had a RIN greater than 7.

The Isolation of poly(A) mRNA from 300 ng of total RNA was achieved using a magnetic isolation module (New England Biolabs, ON, Canada, NEB #E7490) and Invitrogen Magnetic Stand-96, Thermo Fisher Scientific, Cat. #AM10027). RNAseq libraries were constructed using the NEBNext Ultra II RNA library Prep kit for Illumina sequencing (New England Biolabs, NEB #E7770) with the index NEB #E6609. Fragmentation was performed on mRNA for 13 min to obtain fragment size ∼ 300 nucleotides (step 1.2.37, NEB #E7770 protocol). Twelve PCR cycles were used for the PCR enrichment of adaptor-ligated DNA (step 1.9.3, NEB #E7770 protocol). The library quality was evaluated using a DNA 1000 chip on the 2100 Bioanalyzer. Finally, libraries were normalized manually and pooled prior to sequencing on the HiSeq 2500 sequencer using rapid-Run Mode in two lanes of a 2 × 100 bp flow cell at the next generation sequencing platform of the Centre de Recherche du Centre Hospitalier Universitaire de Québec-Université Laval (CHUL, Québec City, QC, Canada). Sequences were deposited in the NCBI Sequence Read Archive under project number PRJNA762091 (accession number SRR15834063 to SRR15834092).

### Mapping and Functional Annotation

The raw sequencing reads were trimmed, filtered and processed for a quality check using CLC Genomics Workbench (CLCBio, QIAGEN).^1^ The adaptors and raw reads with a quality score less than 0.05 (default setting, Phread 13, 95%) were removed. *S. bebbiana* reads were mapped to the closest high-quality genome available (*Salix brachista* C.K. Schneid., GCA_009078335.1_ASM907833v1) using CLCBio. The GenBank format file (*.gbff) was imported into CLCBio (as a track format) to get genomic and coding sequences (CDS) as a reference for the mapping. The number of mapped reads per gene (total exon reads per gene) was stored in the matrix format for further analyses.

### Differential Gene Expression Analysis

The R package DESeq2 was used to determine statistically significant differential expression using a model based on the negative binomial distribution (Love et al., 2014). Outliers were identified by robust principal component analysis (using ROBPCA and GRID algorithms) implemented in the R package rrcov (Chen et al., 2020) and were removed from further analysis. After sequencing, quality control, and data processing steps, each treatment/control contains three to four biological replicates. For the false discovery rate controlling, Benjamini and Hochberg’s approach was implemented (Benjamini and Hochberg, 1995). Thresholds combining false discovery rate (FDR) < 0.05 and the LFC (log2 fold change) >2.0 were used to define significant differentially expressed genes (DEGs). Venn diagrams of differentially expressed genes were generated using InteractiVenn http://www.interactivenn.net/ and other figures were generated with the ggplot2 package in R.

### Functional Analysis

OmicsBox 1.1.11 (Bioinformatics Made Easy, BioBam Bioinformatics, 3 March 2019)^2^ was used for BLAST, GO (Gene Ontology) annotation, Enzyme Code annotation and the Kyoto Encyclopedia of Genes and Genomes (KEGG) mapping of all significantly deregulated transcripts. The Fisher’s exact test (in Blast2GO) was used for the GO enrichment analysis (Götz et al., 2008). The local BLAST has been directed on the plant.193.protein.faa.gz RefSeq database (from NCBI) for the mapping, and the InterPro annotation followed default settings. Transcription factors were identified on PlantTFDB^3^ database using *Salix purpurea* L. (closest available genome in this database) as a reference (Jin et al., 2017).

### Validation of Gene Expression With Real-Time Quantitative PCR

To validate the accuracy of the RNA-Seq expression profiles, eight genes of interest (GOI) from the shared deregulated set of genes (shared among all experiments) identified by transcriptomics were selected for quantitative real-time PCR analysis (Supplementary Table 4; Livak and Schmittgen, 2001; Ahmed and Kim, 2018). These eight GOI (three upregulated and five downregulated) were randomly selected from the list of 40 shared genes (nine upregulated and 31 downregulated) which were differentially expressed in all tailings (CF, CM, and TT). We also selected nine *S. brachista* homologs of the 104 stably expressed genes of *Arabidopsis* (Zhuo et al., 2016) to design primers for normalization of gene expression of *S. bebbiana* samples (Supplementary Table 5). The ReFinder^4^ was used to select the four most stably expressed reference housekeeping genes (HKG-2, HKG-4, HKG-5, HKG-8) in *S. bebbiana* samples (Supplementary Figure 2). The primers designed for GOI primers are listed in Supplementary Table 4 and the primers designed for reference genes are listed in Supplementary Table 5.

The cDNA was synthesized from 200 ng of the total RNA using the Quantitate Reverse Transcription Kit (QIAGEN, Cat. no. 205310). The primers for qPCR were designed using the Oligo Explorer program^5^ (more detail about primer design is provided in the Supplementary Material). No template control reactions were run on each primer pair to detect dimer formation. Gene expression was analyzed for each of the samples using the ABI7500 Fast Real-Time PCR (Applied Biosystems, Thermo Fisher Scientific, Waltham, MA, United States). All reactions were performed in a final volume of 10 μl and contained 1× QuantiTect SYBR Green PCR Master Mix (QIAGEN, Cat. #204145), 0.6 μM of each primer, and 2 μl of template cDNA, which is equivalent to 10 ng of total RNA. PCR thermocycling conditions were set at 95°C for 15 min, 40 cycles at 95°C for 15 s and 65°C for 120 s. Fluorescent readings were taken at the end of each cycle, and the specificity of amplification as well as the absence of primer dimers were confirmed with a melting curve analysis at the end of each reaction. All biological replicates and negative controls were amplified in duplicate. Relative gene expression was calculated using the Double delta CT method (R package “pcr”) after normalization with reference genes (Ahmed and Kim, 2018). The qPCR expression patterns were compared to RNA-Seq normalized expression profiles (More detail is provided in Supplementary Material).

## Results

### Impact of Oil Sands Tailings-Induced Stress on Global Gene Expression of Willow

**FIGURE 2:**
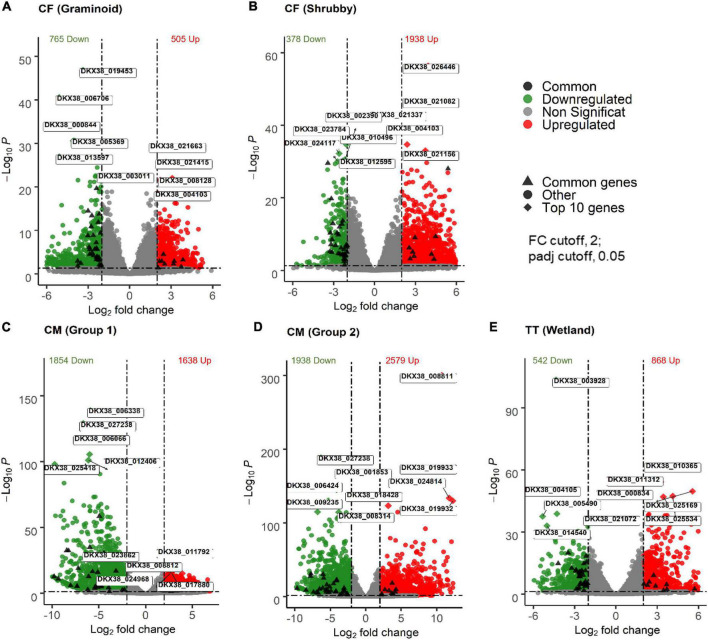
Gene expression of willow exposed to different types of oil sands tailings (OST) stress. Willow grown on CF (with or without capping) along with graminoid **(A)** or shrubby **(B)** plant community. Willow grown along with graminoid fen community on CM with 5 cm capping compared to 90 cm capping **(C)** or 40 cm capping **(D)**. Willow grown on TT tailings (with or without capping) along with wetland plant community **(E)**. Volcano plots show the log transformed adjusted *p*-values of genes plotted on the *y*-axis and log_2_ fold change values on the *x*-axis. Dotted line indicates cut-off of *p*-values and log2 fold change. Top 10 (5 upregulated and 5 downregulated) genes are labeled and functions of theses genes are shown in Figure 3. FC, fold change; Padj, BH-adjusted *p*-values; CF, centrifuge tailings; CM, co-mix tailings; TT, thickened tailings.

RNA sequencing generated an average of 20.92 million high quality reads for all groups of samples, ranging from 13.92 to 25.35 million reads: CF (Graminoid) = 17.94; CF (Shrubby) = 25.35; CM (Group 1) = 13.92; CM (Group2) = 25.16); and TT (Wetland) = 22.21 (Supplementary Table 6). About 90% of total reads were mapped to *S. brachista* (Supplementary Table 6). To analyze variation in gene expression under OST related stresses, DEGs with LFC >2.0 and FDR < 0.05 between treatments in which willows showed signs of OST-related stress (i.e., columns covered with no reclamation cap or 5 cm of reclamation cap) and controls in which willows showed no sign of stress (i.e., columns with ≥30 cm of reclamation cap) were considered significantly expressed. Overall, the samples from each treatment group showed significantly different transcriptomic profiles than samples from the control group (Figure 2 and Supplementary Figure 3). The level of willow gene response was distinct in each type of tailings. The global gene expression analysis of treatments and controls revealed large numbers of DEGs potentially linked with tailings-induced abiotic stresses. The highest number of significant DEGs were identified in CM, followed by CF and TT (Figure 2). In CF, the willows growing within the shrubby fen plant community showed more significant DEGs (1398 upregulated and 378 downregulated) than the willows growing along the graminoid rich fen community (505 upregulated and 765 downregulated). Both groups of CM showed a large number of significant DEGs. In CM (Group 1), which compared the 5 cm PMM/85 cm CM (treatment) to the 5 cm PMM/85 cm till (control), 1638 genes were upregulated and 1854 genes were downregulated, while in CM (Group 2) which compared the 5 cm PMM/85 cm CM (treatment) to the 5 cm PMM/35 cm till/50 cm CM (control), 2579 genes were upregulated and 1938 downregulated due to stress associated with tailings. In TT, 868 genes were upregulated and 542 genes downregulated in the treatment (0 cm PMM) compared to the control (30 cm PMM) (Figure 2).

### Identification of Deregulated Genes in Response to Various Types of Tailings

**FIGURE 3:**
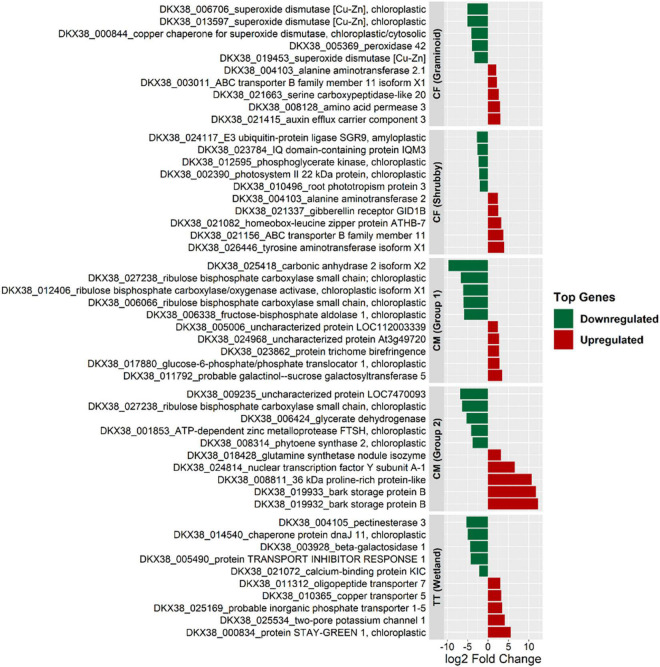
Gene expression and annotation of top 10 highly impacted genes (i.e., with highest log2 fold change in treatments compared to control in each group) of willow grown in different types of oil sands tailings with no reclamation cap or 5 cm of reclamation cap (treatments) compared with ≥30 cm of reclamation cap (controls). CF, centrifuge tailings; CM, co-mix tailings; TT, thickened tailings.

**FIGURE 4:**
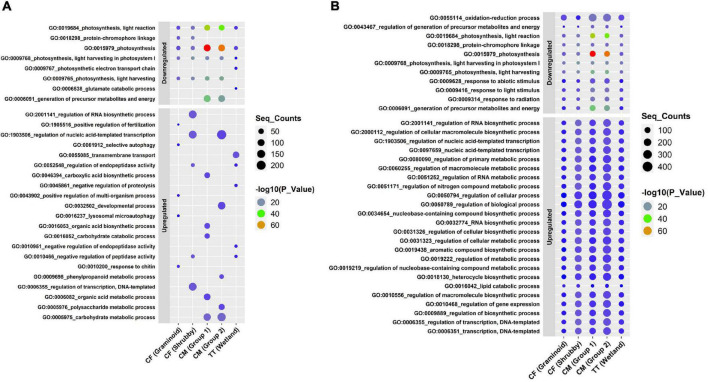
Significantly enriched gene ontology (GO) terms annotated to differentially expressed genes (DEGs) of willow grown in different types of oil sands tailings with no reclamation cap or 5 cm of reclamation cap (treatments) compared with ≥30 cm of reclamation cap (controls). The top 10 highly enriched GO terms (biological process) in each group **(A)** and GO terms (biological process) shared among all groups **(B)** for CF, CM, and TT are shown. Significant enriched genes (*p*-value < 0.05) are colored according to −log10 *p*-value and the dot size indicates the number of sequences annotated with GO term (Seq_Counts). CF, centrifuge tailings; CM, co-mix tailings; TT, thickened tailings.

Willow showed a unique molecular response to each type of tailings as shown by strong (i.e., up to 12 log2 fold change) gene deregulation (Figure 3). In CF tailings, for both the Graminoid and Shrubby groups, genes related to ABC transporter (ABC transporter B family member 11 isoform X1) and plant hormones (auxin and gibberellin) were among the top five upregulated genes. Galactinol-sucrose galactosyltransferase activity was highest in CM (Group 1) in response to the treatment (5 cm PMM/85 cm CM). In CM (Group 2), two highly upregulated genes were related to storage proteins (bark storage protein B) and glutamine synthetase activity. In TT, genes for cellular copper ion and inorganic phosphate transmembrane transporter activity appeared among the highly upregulated genes. Among the highly impacted downregulated genes, about half annotated to the chloroplast structure and functions across all the treatments (Figure 3). Similarly, most of the highly enriched GO terms assigned to upregulated genes were related to catalytic activity and transmembrane transport while highly downregulated genes were related to chloroplast structure (thylakoid, photosynthetic membrane) and functions (mostly related to photosynthesis) GO categories (Figure 4).

### Identification of Shared Genes in Willow Grown in Different Oil Sands Tailings Types

**FIGURE 5:**
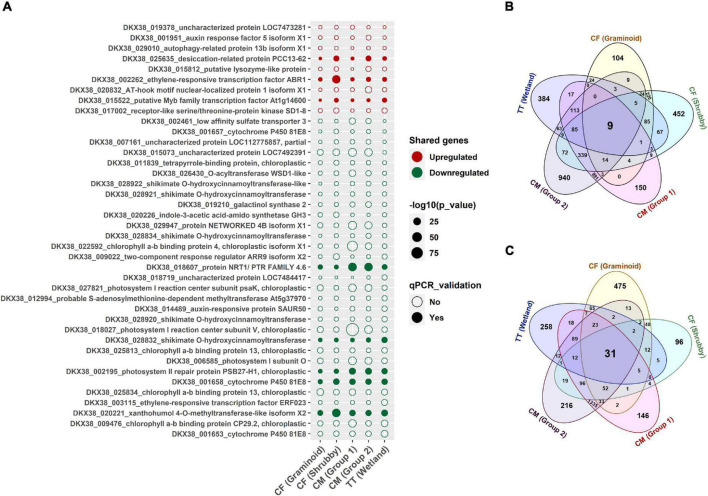
Shared and group specific differentially expressed genes (DEGs) in willow grown in different types of oil sands tailings with no reclamation cap or 5 cm of reclamation cap (treatments) compared with ≥30 cm of reclamation cap (controls). Functions and locus tags of genes which are shared among all groups are shown in dot plot **(A)**. Numbers of significantly upregulated genes that are shared and unique in each group **(B)** and numbers of significantly downregulated genes that are shared and unique in each group **(C)** are shown in Venn diagrams. Only genes with BH-adjusted *p*-values < 0.05 and log_2_ fold change >2 in each group were considered significantly deregulated and the dot size corresponds to the −log10 *p*-value. Genes validated with qPCR are highlighted by filled dots. CF, centrifuge tailings; CM, co-mix tailings; TT, thickened tailings.

To identify potential OST stress-responsive biomarkers in willow, we looked for shared deregulated genes across all treatments of CF, CM and TT. Although plants grown on different OST types showed a distinct profile of transcripts, 40 significantly deregulated (LFC >2.0 and FDR < 0.05) genes were shared among all types of OST treatments. Among these shared genes, nine genes were upregulated and 31 genes were downregulated (Figure 5). Genes related to plant hormones ethylene (ethylene-responsive transcription factor ABR1) and auxin (auxin response factor 5 isoform X1) were among the upregulated transcripts. Moreover, genes related to osmotic adjustment (desiccation-related protein PCC13-62), plant receptor-like serine threonine kinase and MYB family transcription factor were upregulated in all OST treatments. Several upregulated genes were annotated to GO terms related to metabolic process regulation, including regulation of macromolecule metabolic processing. These GO terms were found shared among all types of tailings (Figure 4B).

Many genes linked to chlorophyll (4), photosynthesis (4), shikimate O-hydroxycinnamoyltransferases (5), and cytochrome P450 81E8 (3) were commonly downregulated in willow in response to OST stress. Moreover, several downregulated GO terms (including highly enriched and shared) were related to photosynthesis.

The expression levels of selected genes (three upregulated and five downregulated genes) from the shared set of genes were further validated by qPCR on all the samples. The qPCR results were significantly correlated with RNAseq data from all tailings treatments tested in this experiment (Supplementary Figure 4).

### Identification of Genes Involved in Detoxification of Xenobiotics

**FIGURE 6:**
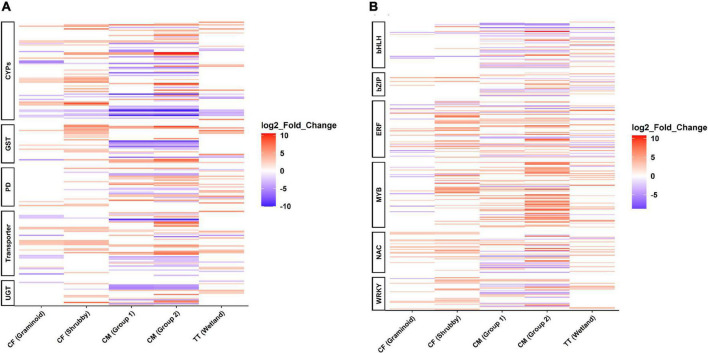
Differentially expressed genes (DEGs) in willow in response to oil sands tailings induced stress. DEGs involved in xenobiotic detoxification pathways **(A)** and in stress-responsive transcription factors **(B)** (known from other studies, Yoon et al., 2020). Red indicates upregulated genes and blue indicates downregulated genes (only significantly deregulated genes LFC > 2, padj < 0.05 are shown in the figure). Cytochrome P450 (CYPs), glutathione S-transferase regulatory process (GST), protein detoxification (PD), ABC transporters (Transporters), UDP-glycosyltransferase (UGT), Basic helix-loop-helix (bHLH), Basic leucine zipper (bZIP), Ethylene-responsive factor (ERF), NAC [derived from no apical meristem (NAM), Arabidopsis transcription activator factor 1/2 (ATAF1/2), and cup-shaped cotyledon 2 (CUC2)], MYB [MYB (myeloblastosis) family of transcription factors]. CF, centrifuge tailings; CM, co-mix tailings; TT, Thickened tailings.

The transcripts linked to three phases of xenobiotic detoxification (transformation, conjugation, and compartmentalization) were deferentially regulated in willows exposed to a variety of OST associated CTEC (PHCs and NAs). Higher numbers of genes related to detoxification (GST, UGTs, CYPs, and ABC transporters) were expressed in the treatment group compared to the control group. Collectively, 151 genes were significantly upregulated for GST (39), UGT (17), CYPs (58), and ABC transporters (37), in response to OST related toxicity (Figure 6A). Moreover, the KEGG pathway “Metabolism of xenobiotics by cytochrome P450” was upregulated in all tailings treatments (Supplementary Figure 5).

### Identification of Genes Involved in Other Abiotic Stresses

Stress-responsive transcription factors like bZIP, BHLH, ERF, MYB, NAC, WRKY were upregulated in all the groups (CF/Graminoid; 34, CF/Shrubby; 86, CM/Group 1; 75, CM/Group2; 114, TT/Wetland; 61). Among the upregulated transcription factors, MYB was the most common (108) followed by ERF (94), NAC (61), WRKY (46), BHLH (35), and bZIP (26) (Figure 6B). Interestingly, an ethylene-responsive factor (ERF), an auxin response factor 5, and a putative MYB family transcription factor were consistently upregulated in all treatments (shared in all groups) (Figure 6B). Moreover, the expression level of the ethylene-responsive factor (DKX38_002262) was validated by qPCR (Supplementary Figure 4).

## Discussion

The results described in this paper indicate that there are both shared and tailings type specific transcriptional responses to OST stress treatments in willow. The shared genes suggest that most of the OST-triggered stress responses are funneled through a small number of signaling and regulatory pathways. The OST-induced stress responses observed were more likely related to the presence of PHCs, NAs, and elevated concentrations of ions (like Ca^2+^, Na^+^, Cl^–^ and HCO_3_^–^) in the tailings (Supplementary Table 1). Although the involvement of multiple pathways cannot be ruled out, the willow transcriptome responses showed that OST induced classes of genes that are commonly involved in xenobiotic detoxification and defense signaling pathways in plants (Ramel et al., 2012). Previous studies showed that genes related to xenobiotic detoxification (GST, UGTs, CYPs, and ABC transporters) and oxidative stress were upregulated after exposure to NAs and PHCs (Gonzalez et al., 2015, 2018; Widdup et al., 2015). Moreover, genes related to three phases of xenobiotic detoxification (transformation, conjugation, and compartmentalization) were deferentially regulated in plants exposed to a variety of CTEC (Ramel et al., 2012; Widdup et al., 2015). We identified several genes and pathways known for detoxification of these compounds that were induced by OST-associated stress.

The transformation phase of xenobiotic detoxification consists of a variety of reactions, including catalysis, oxidation, reduction, and hydrolysis, for the production of intermediate compounds with exposed functional groups, which serve as a substrate for conjugation reactions (Eapen et al., 2007; Abhilash et al., 2009). CYPs are the most common enzyme class involved in transformation of various phytotoxic compounds through a variety of reactions that include hydrolysis, oxidation and reduction. Multiple CYPs were upregulated in the roots and shoots of *Arabidopsis* after exposure to the acid-extractable organic fraction of oil sands process-affected water (Widdup et al., 2015). CYPs were also found upregulated in *Salix viminalis* L. after short exposure (24 h) to PAH (phenanthrene) (Xia et al., 2021). In the conjugation phase, transferases such as GSTs and UGTs participate in the modification of toxicants. We observed an increased transcription level of genes encoding these enzymes in willow exposed to OST. In the compartmentalization phase, conjugates are moved from the cytosol of the cell into the vacuole or apoplast by membrane transporters such as ABC transporters, where they remain stable or are further metabolized. Moreover, genes encoding phenylalanine ammonia-lyase (PAL), which is involved in the first step of the phenylpropanoid pathway, were upregulated in our study. Gonzalez et al. (2015) found PAL as one of the most abundant DEGs in willow grown in PAH contaminated soil.

Other than CTEC, OST may induce responses in willow for multiple abiotic stresses such as osmotic and nutrient deficiency stress. In our experiment, several plant stress-responsive transcription factors (e.g., bZIP, BHLH, ERF, MYB, NAC, WRKY) were upregulated by OST stress. Interestingly, ARF (auxin response factor), putative MYB family transcription factor, and ERF (ethylene-responsive transcription factor ABR1) were commonly upregulated in response to all treatments. Moreover, the expression level of ethylene-responsive transcription factor ABR1 was also validated during our qPCR experiment. Hormonal involvement in response to abiotic stress, including xenobiotic stress, was reported previously (Ramel et al., 2007; Weisman et al., 2010). The stress hormone abscisic acid (ABA) regulates plant abiotic stress responses by activating target transcription factors, such as the basic leucine zipper (bZIP; Ramel et al., 2007; Zong et al., 2016), and JA signaling pathways activate the basic helix-loop-helix (bHLH) transcription factors (Goossens et al., 2017). These transcription factors regulate the expression of stress-responsive genes, as demonstrated in several studies (Zong et al., 2016; Goossens et al., 2017; Joo et al., 2019). Many other stress-responsive transcription factors such as ERF, MYB, NAC, and WRKY have been identified by various experimental approaches (Yoon et al., 2020).

The ERF (Ethylene-responsive factor) is involved in meditating plant tolerance to several abiotic stresses. Numerous reports have shown that ERFs are critical in ABA-dependent stress responses (Hsieh et al., 2013; Chen et al., 2016; Yao et al., 2017). During plant responses to abiotic stresses, MYBs tend to interact with other stress-responsive transcription factors, such as bHLH and WRKYs, which support the finding that MYBs are involved both directly and indirectly in plant tolerance to abiotic stresses (Feller et al., 2011; Wang et al., 2015; Yu et al., 2016). The expression patterns of NACs and WRKYs gene family members are regulated by abiotic stress. Jiang and Deyholos (2006) found that 33 NAC and 18 WRKYs genes were significantly upregulated by salt stress in *Arabidopsis*. Similarly, NACs and WRKYs were also upregulated under PAH stress (Weisman et al., 2010; Widdup et al., 2015). The striking influence on photosynthetic gene expression suggests that OST stress-induced photosystem I (PSI) and photosystem II (PSII) perturbation and oxidative stress is coordinated along with a cascade of a regulatory networks involving metabolic and hormonal signaling pathways, as was reported previously during PAH stress (Liu et al., 2009; Weisman et al., 2010). Our results are consistent with the various studies related to plant responses to xenobiotics. However, considering the significant differences in plant growth between control and treated plants, a leaf age-related sampling bias cannot be ruled out entirely. For instance, it has been recently reported that transcriptomic and metabolic profiles can vary with the age of leaves (Chang et al., 2020). Moreover, toxic compounds can accumulate over time in plant tissues (Simonich and Hites, 1995), which could have further increased differences in gene expression between plants grown under control and treatment conditions. Although here RNAseq was performed on fully developed leaves at similar physiological stage (based on visual observations) to circumvent sampling variations, the use of other strategies allowing to better estimating the age of leaves, such as the leaf plastochron index (Meicenheimer, 2014), should be considered in future studies.

Overall, these results showed that willow plants grown directly on OST were likely under multiple OST-associated stresses, altering their physiological and transcriptomics responses. However, capping OST with either PMM or till helped to mitigate the level of OST stress (Supplementary Figure 1), probably by diluting the effect of CTEC and salts in OST or by limiting root contact with the tailings. Reclamation cap placed on tailings can facilitate vegetation establishment by providing the resources for root growth and anchorage including nutrients, moisture, porosity and aeration. Capping materials can also be a source of beneficial root-associated microorganisms that may have contributed to plant growth and reduction of environmental stress through various mechanisms such as enhanced nutrient cycling and uptake, phytohormones production, or contaminants degradation (Correa-García et al., 2018). The contrasting willow responses due to different accompanying plant communities (graminoid vs. shrubby in CF) are also most likely linked with complex root-level interactions (including root-microorganisms interactions), which indicate that selection of plant species is important to modulate the responses to OST-associated stress. Further studies allowing for the monitoring of root development and the characterization of root-associated microbial communities will be needed to decipher the belowground interactions underlying these observations. The shared (40 genes including four transcription factors) and validated set of genes (eight genes) identified here can ultimately provide tools to facilitate the achievement of such studies, but can also become the building blocks to develop a diagnostic approach to stress response in willow and related species exposed to OST. These results provide a large number of new pathway targets and plant biosensors for OST toxicity assessment which may assist in guiding the design of OST reclamation treatments aimed at the rapid restoration of mine disturbed sites.

## Data Availability Statement

The datasets presented in this study can be found in online repositories. The names of the repository/repositories and accession number(s) can be found below: https://www.ncbi.nlm.nih.gov/, PRJNA762091.

## Author Contributions

CM, ASé, DD, and DM contributed to the conception and design of study. DD conducted greenhouse experiments. ASa and GP carried out lab experiments and performed bioinformatics analysis. ASa prepared figures and wrote the manuscript. All authors contributed to manuscript revision, read, and approved the submitted version.

## Conflict of Interest

The authors declare that the research was conducted in the absence of any commercial or financial relationships that could be construed as a potential conflict of interest.

## Publisher’s Note

All claims expressed in this article are solely those of the authors and do not necessarily represent those of their affiliated organizations, or those of the publisher, the editors and the reviewers. Any product that may be evaluated in this article, or claim that may be made by its manufacturer, is not guaranteed or endorsed by the publisher.

## Footnotes

1
http://www.clcbio.com

2
https://www.biobam.com/omicsbox

3
http://planttfdb.gao-lab.org/index.php?sp=Spu

4
https://www.heartcure.com.au/reffinder/?type=reference

5
http://www.genelink.com/tools/gl-oe.asp

## References

[B1] Abhilash P. C. Jamil S. Singh N. (2009). Transgenic plants for enhanced biodegradation and phytoremediation of organic xenobiotics. *Biotechnol. Adv.* 27 474–488. 10.1016/j.biotechadv.2009.04.002 19371778

[B2] Ahmed M. Kim D. R. (2018). Pcr: an R package for quality assessment, analysis and testing of qPCR data. *PeerJ* 6:e4473. 10.7717/peerj.4473 29576953 PMC5858653

[B3] Argus G. W. (2007). Salix (Salicaceae) distribution maps and a synopsis of their classification in North America, North of Mexico. *Harv. Pap. Bot.* 12 335–368. 10.3100/1043-4534(2007)12[335:ssdmaa]2.0.co;2

[B4] Azam S. Rima U. S. (2014). Oil sand tailings characterisation for centrifuge dewatering. *Environ. Geotech.* 1 189–196. 10.1680/envgeo.13.00044

[B5] Badiozamani M. M. Askari-Nasab H. (2014). Integration of reclamation and tailings management in oil sands surface mine planning. *Environ. Model. Softw.* 51 45–58. 10.1016/j.envsoft.2013.09.026

[B6] Benjamini Y. Hochberg Y. (1995). Controlling the false discovery rate: a practical and powerful approach to multiple testing. *J. R. Stat. Soc. Ser. B Methodol.* 57 289–300. 10.1111/j.2517-6161.1995.tb02031.x

[B7] BGC Engineering Inc (2010). *Oil Sands Tailings Technology Review.* Edmonton, AB: University of Alberta Libraries. 10.7939/R3Z60C18G

[B8] Chalaturnyk R. J. Scott J. D. Özüm B. (2002). Management of oil sands tailings. *Pet. Sci. Technol.* 20 1025–1046. 10.1081/LFT-120003695

[B9] Chang W. Zhao H. Yu S. Yu J. Cai K. Sun W. (2020). Comparative transcriptome and metabolomic profiling reveal the complex mechanisms underlying the developmental dynamics of tobacco leaves. *Genomics* 112 4009–4022. 10.1016/j.ygeno.2020.07.005 32650092

[B10] Chen H.-Y. Hsieh E.-J. Cheng M.-C. Chen C.-Y. Hwang S.-Y. Lin T.-P. (2016). *ORA47* (*octadecanoid-responsive AP2/ERF-domain transcription factor 47*) regulates jasmonic acid and abscisic acid biosynthesis and signaling through binding to a novel cis-element. *New Phytol.* 211 599–613. 10.1111/nph.13914 26974851

[B11] Chen X. Zhang B. Wang T. Bonni A. Zhao G. (2020). Robust principal component analysis for accurate outlier sample detection in RNA-Seq data. *BMC Bioinformatics* 21:269. 10.1186/s12859-020-03608-0 32600248 PMC7324992

[B12] Correa-García S. Pande P. Séguin A. St-Arnaud M. Yergeau E. (2018). Rhizoremediation of petroleum hydrocarbons: a model system for plant microbiome manipulation. *Microb. Biotechnol*. 11 819–832. 10.1111/1751-7915.13303 30066464 PMC6116750

[B13] Eapen S. Singh S. D’Souza S. F. (2007). Advances in development of transgenic plants for remediation of xenobiotic pollutants. *Biotechnol. Adv.* 25 442–451. 10.1016/j.biotechadv.2007.05.001 17553651

[B14] Feller A. Machemer K. Braun E. L. Grotewold E. (2011). Evolutionary and comparative analysis of *MYB* and *bHLH* plant transcription factors. *Plant J.* 66 94–116. 10.1111/j.1365-313X.2010.04459.x 21443626

[B15] Gonzalez E. Brereton N. J. B. Marleau J. Guidi Nissim W. Labrecque M. Pitre F. E. (2015). Meta-transcriptomics indicates biotic cross-tolerance in willow trees cultivated on petroleum hydrocarbon contaminated soil. *BMC Plant Biol.* 15:246. 10.1186/s12870-015-0636-9 26459343 PMC4603587

[B16] Gonzalez E. Pitre F. E. Pagé A. P. Marleau J. Guidi Nissim W. St-Arnaud M. (2018). Trees, fungi and bacteria: tripartite metatranscriptomics of a root microbiome responding to soil contamination. *Microbiome* 6:53. 10.1186/s40168-018-0432-5 29562928 PMC5863371

[B17] Goossens J. Mertens J. Goossens A. (2017). Role and functioning of *bHLH* transcription factors in jasmonate signalling. *J. Exp. Bot.* 68 1333–1347. 10.1093/jxb/erw440 27927998

[B18] Götz S. García-Gómez J. M. Terol J. Williams T. D. Nagaraj S. H. Nueda M. J. (2008). High-throughput functional annotation and data mining with the Blast2GO suite. *Nucleic Acids Res.* 36 3420–3435. 10.1093/nar/gkn176 18445632 PMC2425479

[B19] Hargreaves J. Lock A. Beckett P. Spiers G. Tisch B. Lanteigne L. (2012). Suitability of an organic residual cover on tailings for bioenergy crop production: a preliminary assessment. *Can. J. Soil Sci.* 92 203–211. 10.4141/cjss2010-056

[B20] Hsieh E.-J. Cheng M.-C. Lin T.-P. (2013). Functional characterization of an abiotic stress-inducible transcription factor *AtERF53* in *Arabidopsis thaliana*. *Plant Mol. Biol.* 82 223–237. 10.1007/s11103-013-0054-z 23625358

[B21] Huang M. Barbour S. L. Carey S. K. (2015). The impact of reclamation cover depth on the performance of reclaimed shale overburden at an oil sands mine in Northern Alberta, Canada. *Hydrol. Process.* 29 2840–2854. 10.1002/hyp.10229

[B22] Jiang Y. Deyholos M. K. (2006). Comprehensive transcriptional profiling of NaCl-stressed *Arabidopsis* roots reveals novel classes of responsive genes. *BMC Plant Biol.* 6:25. 10.1186/1471-2229-6-25 17038189 PMC1621065

[B23] Jin J. Tian F. Yang D.-C. Meng Y.-Q. Kong L. Luo J. (2017). PlantTFDB 4.0: toward a central hub for transcription factors and regulatory interactions in plants. *Nucleic Acids Res.* 45 D1040–D1045. 10.1093/nar/gkw982 27924042 PMC5210657

[B24] Joo J. Lee Y. H. Song S. I. (2019). *OsbZIP42* is a positive regulator of ABA signaling and confers drought tolerance to rice. *Planta* 249 1521–1533. 10.1007/s00425-019-03104-7 30712129

[B25] Kasperski K. L. (1992). A review of properties and treatment of oil sands tailings. *AOSTRA J. Res.* 8 11–53. 10.1016/j.chemosphere.2015.02.003 25753852

[B26] Kuzovkina Y. A. Quigley M. F. (2005). Willows beyond wetlands: uses of *Salix L.* species for environmental projects. *Water. Air. Soil Pollut.* 162 183–204. 10.1007/s11270-005-6272-5

[B27] Kuzovkina Y. A. Volk T. A. (2009). The characterization of willow (*Salix L.*) varieties for use in ecological engineering applications: co-ordination of structure, function and autecology. *Ecol. Eng.* 35 1178–1189. 10.1016/j.ecoleng.2009.03.010

[B28] Lalonde R. S. Pinno B. D. MacKenzie M. D. Utting N. (2020). Capping dewatered oil sands fluid fine tailings with salvaged reclamation soils at varying depths to grow woody plants. *Can. J. Soil Sci.* 100 546–557. 10.1139/cjss-2019-0120

[B29] Li X. Ma B. Drozdowski B. Salifu F. Chang S. X. (2020). Effects of capping strategy and water balance on salt movement in oil sands reclamation soils. *Water* 12:512. 10.3390/w12020512

[B30] Limited H. B. (1989). *Manual of Plant Species Suitability for Reclamation in Alberta −. Alberta Land Conservation and Reclamation Council Report No. RRTAC 89-4*, 2nd Edn. Edmonton, AB: Alberta Land Conservation and Reclamation Council, Reclamation Research Technical Advisory Committee. 10.7939/R3FW17

[B31] Liu H. Weisman D. Ye Y. Cui B. Huang Y. Colón-Carmona A. (2009). An oxidative stress response to polycyclic aromatic hydrocarbon exposure is rapid and complex in *Arabidopsis thaliana*. *Plant Sci.* 176 375–382. 10.1016/j.plantsci.2008.12.002

[B32] Livak K. J. Schmittgen T. D. (2001). Analysis of relative gene expression data using real-time quantitative PCR and the 2^–ΔΔ*CT*^ method. *Methods* 25 402–408. 10.1006/meth.2001.1262 11846609

[B33] Love M. I. Huber W. Anders S. (2014). Moderated estimation of fold change and dispersion for RNA-seq data with DESeq2. *Genome Biol.* 15:550. 10.1186/s13059-014-0550-8 25516281 PMC4302049

[B34] MacKenzie D. J. McLean M. A. Mukerji S. Green M. (1997). Improved RNA extraction from woody plants for the detection of viral pathogens by reverse transcription-polymerase chain reaction. *Plant Dis.* 81 222–226. 10.1094/PDIS.1997.81.2.222 30870901

[B35] Meicenheimer R. D. (2014). The plastochron index: still useful after nearly six decades. *Am. J. Bot*. 101 1821–1835. 10.3732/ajb.1400305 25366849

[B36] Mosseler A. Major J. E. Labrecque M. (2014). Growth and survival of seven native willow species on highly disturbed coal mine sites in eastern Canada. *Can. J. For. Res.* 44 340–349. 10.1139/cjfr-2013-0447

[B37] Parrott J. L. Raine J. C. McMaster M. E. Hewitt L. M. (2019). Chronic toxicity of oil sands tailings pond sediments to early life stages of fathead minnow (*Pimephales promelas*). *Heliyon* 5:e02509. 10.1016/j.heliyon.2019.e02509 31687598 PMC6819858

[B38] Ramel F. Sulmon C. Cabello-Hurtado F. Taconnat L. Martin-Magniette M.-L. Renou J.-P. (2007). Genome-wide interacting effects of sucrose and herbicide-mediated stress in *Arabidopsis thaliana*: novel insights into atrazine toxicity and sucrose-induced tolerance. *BMC Genom.* 8:450. 10.1186/1471-2164-8-450 PMC2242805 18053238

[B39] Ramel F. Sulmon C. Serra A.-A. Gouesbet G. Couée I. (2012). Xenobiotic sensing and signalling in higher plants. *J. Exp. Bot.* 63:3999–4014. 10.1093/jxb/ers102 22493519

[B40] Simonich S. L. Hites R. A. (1995). Organic pollutant accumulation in vegetation. *Environ. Sci. Technol*. 29 2905–2914. 10.1021/es00012a004 22148195

[B41] Timoney K. P. Ronconi R. A. (2010). Annual bird mortality in the bitumen tailings ponds in northeastern Alberta, Canada. *Wilson J. Ornithol.* 122 569–576. 10.1676/09-181.1

[B42] Wang C. Harbottle D. Liu Q. Xu Z. (2014). Current state of fine mineral tailings treatment: a critical review on theory and practice. *Miner. Eng.* 58 113–131. 10.1016/j.mineng.2014.01.018

[B43] Wang F. Chen H.-W. Li Q.-T. Wei W. Li W. Zhang W.-K. (2015). *GmWRKY27* interacts with *GmMYB174* to reduce expression of *GmNAC29* for stress tolerance in soybean plants. *Plant J.* 83 224–236. 10.1111/tpj.12879 25990284

[B44] Weisman D. Alkio M. Colón-Carmona A. (2010). Transcriptional responses to polycyclic aromatic hydrocarbon-induced stress in *Arabidopsis Thaliana* reveal the involvement of hormone and defense signaling pathways. *BMC Plant Biol.* 10:59. 10.1186/1471-2229-10-59 20377843 PMC2923533

[B45] Widdup E. E. Chatfield-Reed K. Henry D. Chua G. Samuel M. A. Muench D. G. (2015). Identification of detoxification pathways in plants that are regulated in response to treatment with organic compounds isolated from oil sands process-affected water. *Chemosphere* 139 47–53. 10.1016/j.chemosphere.2015.05.048 26052061

[B46] Wolter G. L. L. Naeth M. A. (2014). Dry mature fine tailings as oil sands reclamation substrates for three native grasses. *J. Environ. Qual.* 43 1510–1516. 10.2134/jeq2013.10.0415 25603099

[B47] Xia L. Xiaodong M. Yunhe C. Junxiang L. Junzhu Z. Feifei Z. (2021). Transcriptomic and metabolomic insights into the adaptive response of *Salix viminalis* to phenanthrene. *Chemosphere* 262:127573. 10.1016/j.chemosphere.2020.127573 32745791

[B48] Yao Y. He R. J. Xie Q. L. Zhao X. H. Deng X. M. He J. B. (2017). ETHYLENE RESPONSE FACTOR 74 (*ERF74*) plays an essential role in controlling a respiratory burst oxidase homolog D (RbohD)-dependent mechanism in response to different stresses in *Arabidopsis*. *New Phytol.* 213 1667–1681. 10.1111/nph.14278 28164334

[B49] Yoon Y. Seo D. H. Shin H. Kim H. J. Kim C. M. Jang G. (2020). The role of stress-responsive transcription factors in modulating abiotic stress tolerance in plants. *Agronomy* 10:788. 10.3390/agronomy10060788

[B50] Yu Y.-T. Wu Z. Lu K. Bi C. Liang S. Wang X.-F. (2016). Overexpression of the MYB transcription factor *MYB28* or *MYB99* confers hypersensitivity to abscisic acid in *Arabidopsis*. *J. Plant Biol.* 59 152–161. 10.1007/s12374-016-0463-z

[B51] Zhuo B. Emerson S. Chang J. H. Di Y. (2016). Identifying stably expressed genes from multiple RNA-Seq data sets. *PeerJ* 4:e2791. 10.7717/peerj.2791 28028467 PMC5178351

[B52] Zong W. Tang N. Yang J. Peng L. Ma S. Xu Y. (2016). Feedback regulation of ABA signaling and biosynthesis by a bZIP transcription factor targets drought-resistance-related genes. *Plant Physiol.* 171 2810–2825. 10.1104/pp.16.00469 27325665 PMC4972276

