# Supplementary material for: Understanding Willow Transcriptional Response in the Context of Oil Sands Tailings Reclamation
Source: Front Plant Sci. 2022 Apr 27;13:857535. doi: 10.3389/fpls.2022.857535 (PMC9094116; doi:10.3389/fpls.2022.857535)
Supplement: Supplementary file 1 [file Data_Sheet_1.PDF]

Supplementary Material

1. Primer Design and qPCR

1.1. Primer Design

Primers were designed using the Oligo Analyzer/Oligo Explorer program (http://www.genelink.com/tools/gl-oe.asp). Annealing temperature (Tm) was set at 65°C using the following parameters (salt concentration 50mM and 250pM DNA). Primers specific to target sequences were designed from homologous alignments (*Populus trichocarpa*, *Vitis vinifera*, *Arabidopsis thaliana* and closest species) retrieved from Plaza (https://bioinformatics.psb.ugent.be/plaza/versions/plaza_v2_5/). To these sequences, we added coding sequences (CDS) of *Salix brachista* and contigs from this study obtained from the CLCBio RNASeq analysis. The selected primers were submitted for a BLAST analysis on the NCBI reference database for plant and willow transcriptome to ensure that they match to specific target sequences. The primers were designed within the limits of a single exon, excluding any intron sequence.

Reference genes (House Keeping Gene, HKG) were selected from a list of the 104 most stable gene from *Arabidopsis taliana* (https://peerj.com/articles/2791/#supplemental-information) considering protein similarity and RPKM abundance, stability and function. All set of HKG primers were designed and tested on the all samples. Finally, eight genes were selected based on their stability as described by (Vandesompele et al., 2002). GeNorm Excel Applet (https://genorm.cmgg.be) as well as NormqPCR (https://www.bioconductor.org/packages/release/bioc/html/NormqPCR.html) were used to confirm the stability.

1.2 Reverse Transcription and qPCR

Total RNA (4 µl of a 50 ng/µl dilution) was reverse transcribed using the QuantiTect Reverse Transcription (RT) Kit (Qiagen, Cat. #205310). Resulting cDNA was diluted to a final concentration of 5ng/µl using 10mM Tris, pH 8.0, and stored into separate tubes to avoid repeated freeze/thaw cycles. No RT control reactions were performed for all samples.

To determine efficiencies and specificities of the primer sets, qPCR reactions were carried out at various annealing temperatures using cDNA as template on Applied Biosystems 7500 Real-Time PCR Systems. Once adequate annealing temperature was determined, an initial 15 min activation step at 95°C followed by 40 cycles of PCR were performed using the following amplification conditions; (94°C, 5sec; 65°C, 120sec). Each reaction consisted of 0.6µM of both forward and reverse primers, 1ng of cDNA and 1X QuantiTect SYBR green mix (Qiagen, Cat.204145) in a final volume of 10µl. Fluorescent readings were taken at the end of each cycle and the specificity of amplification as well as absence of primer dimers were confirmed with a melting curve analysis at the end of each reaction. No Template Control (ntc) and no RT control reactions were run together with the samples to check for primers dimerization or DNA contamination.

1.3 qPCR data analysis

The number of molecules initially introduced in the qPCR reaction (No) is evaluated by the No-Ct-Linear Regression of Efficiency (LRE) approach (Rutledge and Stewart, 2008). Individual amplification efficiency is established and applied to the No determination. To reduce the impact of biological variation and technical variation associated with the various steps (RNA extraction, RNA quantification, reverse transcription and RT-qPCR reactions (e.g. inhibitors of PCR)), each No was normalised against the average No of five HKG. This normalized fold change was established by (delta delta Ct) (Livak and Schmittgen, 2001).

References

Livak, K. J., and Schmittgen, T. D. (2001). Analysis of Relative Gene Expression Data Using Real-Time Quantitative PCR and the $2^{-\Delta\Delta CT}$ Method. *Methods* 25, 402–408. doi:10.1006/meth.2001.1262.

Rutledge, R. G., and Stewart, D. (2008). Critical evaluation of methods used to determine amplification efficiency refutes the exponential character of real-time PCR. *BMC Mol. Biol.* 9, 96. doi:10.1186/1471-2199-9-96.

Vandesompele, J., De Preter, K., Pattyn, F., Poppe, B., Van Roy, N., De Paepe, A., et al. (2002). Accurate normalization of real-time quantitative RT-PCR data by geometric averaging of multiple internal control genes. *Genome Biol.* 3, research0034.1. doi:10.1186/gb-2002-3-7-research0034.

2. Supplementary Figures

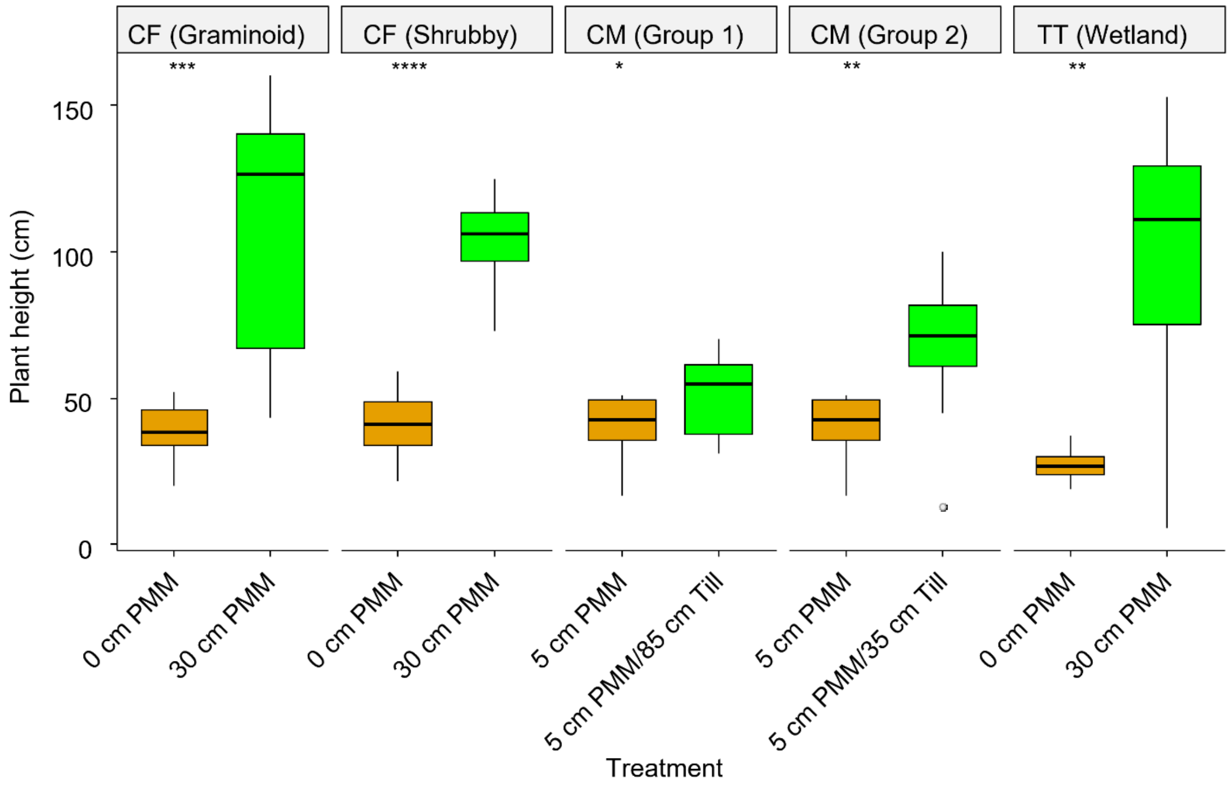

Figure S1. Plant height of willows growing in different tailings and capping treatments (n=4). Willow grown on CF (with or without capping) along with graminoid (A) and shrubby (B) plant community. Willow grown on CM with 5 cm capping or with 90 (C) or 40 (D) cm capping along with graminoid fen community. Willow grown on TT tailings (with or without capping) along with wetland plant community (E). Welch's t-test was used to compare plant height between treatment and control in each group. P-value is indicated by asterisks (*), *<0.05, **<0.005, ***<0.0005, ****<0.00005.

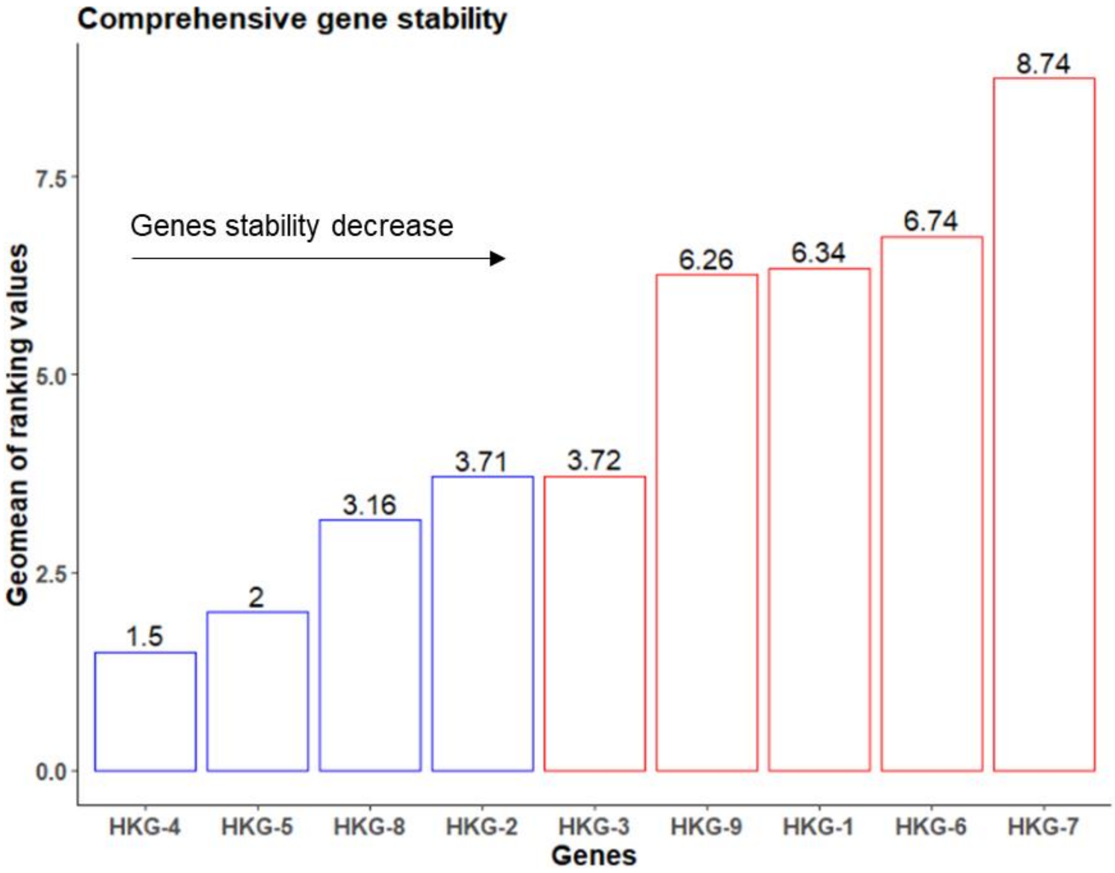

Figure S2. Selection of Reference Genes: Reference genes are ranked based on expression stability calculated by RefFinder, which integrates the results from four different algorithms (geNorm, NormFinder, comparative ΔCq and BestKeeper) and ranks the genes according to their expression stability (https://www.heartcure.com.au/reffinder/?type=reference). Stability values represent the average pairwise variation of the gene compared with all genes analyzed under the same experimental conditions. The analysis was performed using expression data (n = 24). Four genes with lowest geomean value (in blue) were selected for RNAseq data normalization.

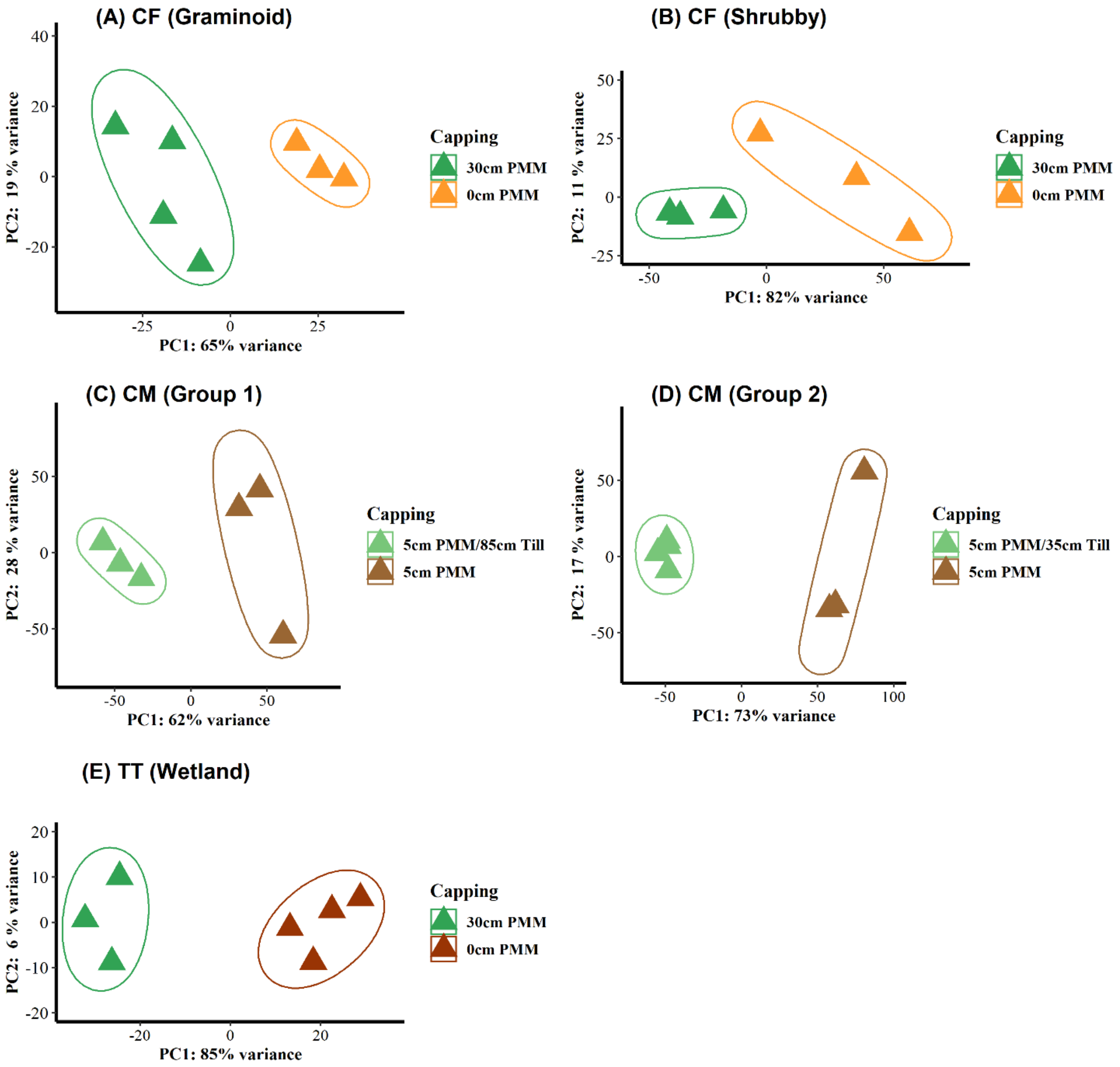

Figure S3. Principal components analysis of transcriptomic data from leaves of willows growing in different tailings and capping treatments. Willow grown on CF (with or without capping) along with graminoid (A) and shrubby (B) plant community. Willow grown on CM with 5 cm capping or with 90 (C) or 40 (D) cm capping along with graminoid fen community. Willow grown on TT tailings (with or without capping) along with wetland plant community.

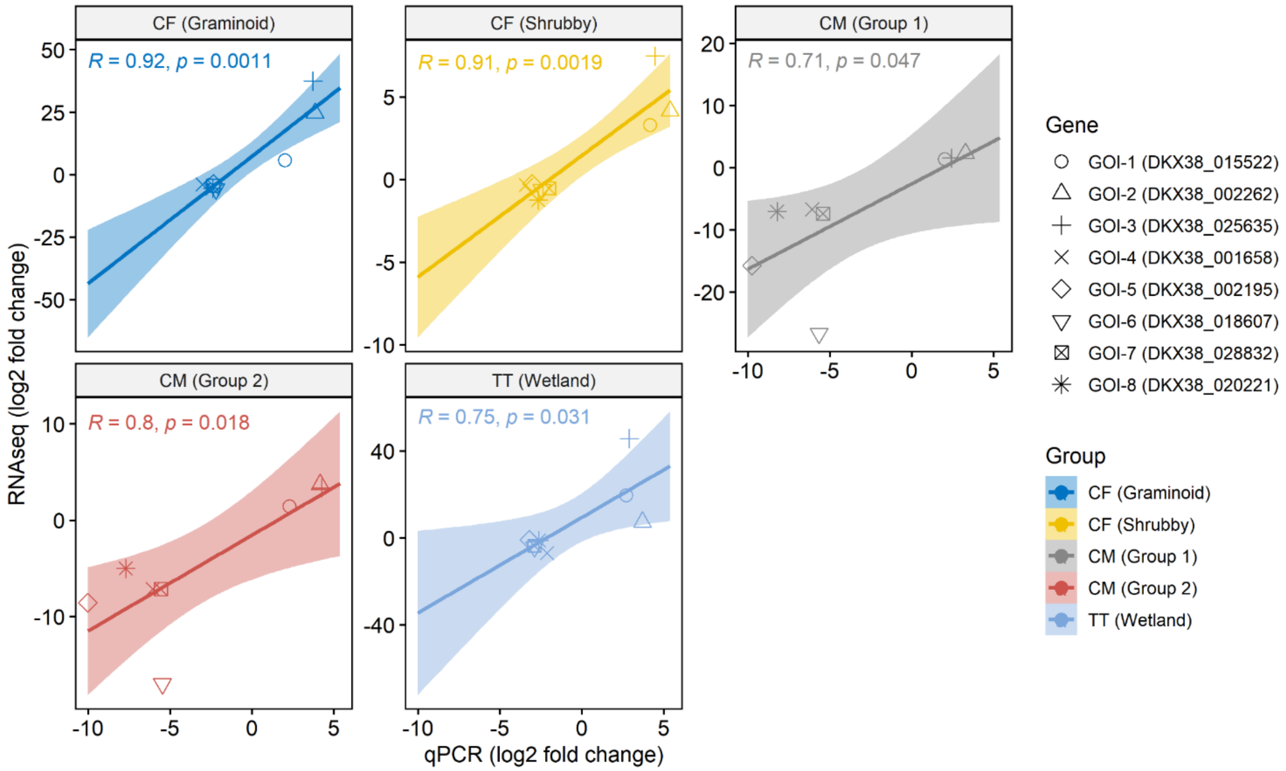

Figure S4. Gene expression correlation between qPCR and RNAseq data of willow grown in different types of oil sands tailings with or without capping material. The Pearson correlation was calculated between selected genes of interest (GOI) which are shared between all group based on RNAseq dataset. The qPCR fold change was calculated using the 'pcr' package in R and RNAseq fold change was calculated in Deseq2.

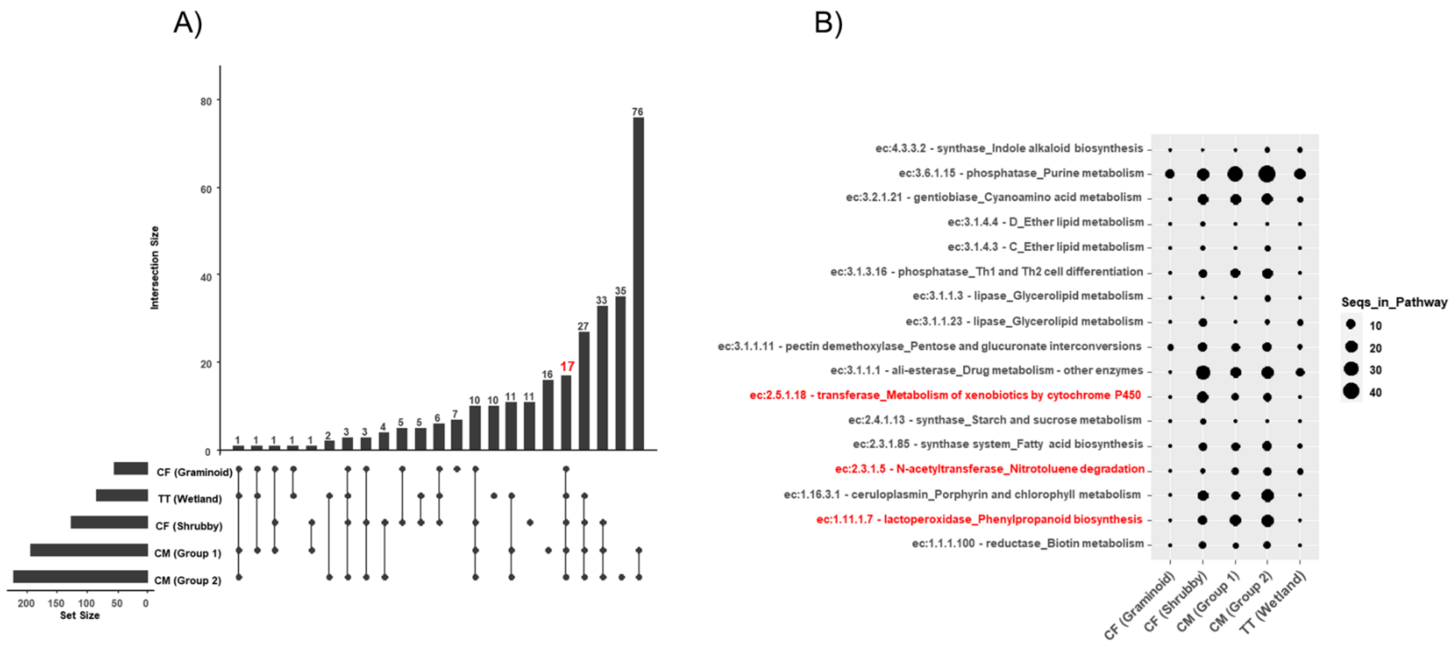

Figure S5. KEGG pathways analysis of significantly upregulated genes considering threshold LFC >2, padj < 0.05. Upset plot shows that 17 KEGG enzymes are shared (highlited in red) in all groups of willow (CF (Graminoid), CF (Shrubby), CM (Group 1), CM (Group 2), TT (Wetland)) (A). Functions of 17 shared enzymes are shown in dot plot and enzymes related to xenobiotic detoxification are highlighted in red (B).

3. Supplementary Tables (Table 1-6)

Table S1. Basic physicochemical properties, plant available nutrients, trace metal concentrations, and organic constituent concentrations of centrifuge (CF), co-mix (CM) and thickened tailings (TT); mean ± one standard deviation of the mean.

Physicochemical properties of tailings	CF	CM	TT
pH	7.8±0.1	7.6±0.1	8.7±0.1
EC (dS/m)	2.0±0.3	3.5±0.2	1.0±0.1
SAR	4.3±0.3	5.3±0.6	1.9±0.1
CEC (cmol/kg)	19±8	19±6	3±1
Solids (%)	59±1.7	66±3	80±2
% clay from MBI	56±6	89±7	18±3
MBI (meq/100 g)	7.8±0.7	12.5±0.9	2.5±0.5
SFR	0.44±0.1	0.55±0.1	2.8±0.5
% particle passing 45 µm by Sieving†	62±6	69±6	27±3
% sand (2 mm – 50 µm) by Hydrometer‡	18±2	26±2	70±7
% silt (2 – 50 µm) by Hydrometer‡	79±1	37±3	17±3
% clay (<2 µm) by Hydrometer‡	3±1	37±1	13±5
% moisture at FC (1 bar)	16±1	22±1	9±2
% moisture at WP (15 bar)	11±0.3	13±0.5	3.9±1
% AWHC	5±1	9±1	5±1
Exchangeable ions and plant available nutrients			
Ca^{2+} (mg/kg)	2,783±670	3,699±411	333±52
K^{+} (mg/kg)	134±26	194±26	48±6
Na^{+} (mg/kg)	941±168	1,396±144	157±17
Mg^{2+} (mg/kg)	489±99	826±119	106±17
Extractable P (mg/kg)	6±6	8±2	2±0
Extractable S (mg/kg)	235±144	403±82	72±7
NO_3-N	38±4	32±9	3±1
NH_4-H	64±14	24±3	10±1
Major water soluble ions			
Ca^{2+} (mg/kg)	771±205	266±119	57±13
Na^{+} (mg/kg)	2,656±207	1,211±112	151±19
K^{+} (mg/kg)	94±13	58±7	22±3
Mg^{2+} (mg/kg)	245±61	96±36	26±6
SO_4^{2-} (mg/kg)	7,287±1236	2,050±836	492±80
Cl (mg/kg)	319±14	382±30	9±1
HCO_3^{-} (mg/kg)	1,226±248	1,305±469	62±42
Trace metal concentrations			
B (mg/kg)	8.8±1	2.6±3	1.0±0.2
Ba (mg/kg)	1.8±0.1	2.2±0.8	0.1±0.01

Cu (mg/kg)	0.11±0.03	0.06±0.02	0.02±0.003
Fe (mg/kg)	0.5±0.2	7.4±7	0.02±0.009
Mn (mg/kg)	5.7±2	0.5±1.4	1.2±0.7
Sr (mg/kg)	17±6	1.6±4	1.1±0.2
Zn (mg/kg)	0.9±0.1	0.5±0.3	<0.012
Organic constituent concentrations			
Benzene (mg/kg)	1.5±0.4	0.3±0.1	<DL
Toluene (mg/kg)	0.1±0.1	0.1±0.1	<DL
Ethylbenzene (mg/kg)	0.2±0.1	2.4±0.5	<DL
Xylene (mg/kg)	2.1±1.2	8±1.8	<DL
F1 C6-C10 (mg/kg)	4,279±1,423	581±114	<DL
F2c C11-C16 (mg/kg)	8,331±1,171	570±114	521±197
F3c C17-C34 (mg/kg)	41,938±5,861	1,357±213	3,688±1,316
F4 C35-C50+ (mg/kg)	37,838±6,406	2,757±407	1,862±673
F5 (mg/kg)	19,900±4,598	2,907±756	1,816±667
NAs (mg/kg)	3,717±770	321±80	227±85
Bitumen (%)	4.8±1.0	0.5±0.2	0.61±0.2

†Samples were bitumen free and done after Dean Stark procedure.

‡Sample preparation to burn off the bitumen was necessary prior to texture analysis by hydrometer method.

AWHC – Available water holding capacity, CEC – Cation exchange capacity, EC – Electrical conductivity, FC – Field capacity, Kc – Clearwater saline-sodic shale overburden, kPa – Kilopascals, MBI – Methylene blue index, NAs – Naphthenic acids, pH – Acidity or alkalinity, SAR – Sodium absorption ratio, SFR – Sand to fine ratio, TOC – Total organic carbon, TN – Total nitrogen, CF – Centrifuge tailings, CM – Co-mix tailings, TT – Thickened tailings.

Table S2. Basic physicochemical properties of capping material, till, and peat mineral mix (PMM) from Syncrude Canada, and Imperial Oil Ltd.; mean ± one standard deviation of the mean.

Physicochemical properties	Till	Syncrude PMM	Imperial PMM
pH	8.1±0.4	6.2±0.4	6.9±0.2
EC (dS/m)	0.4±0.1	0.2±0.1	0.5±0.1
SAR	0.8±0.1	0.4±0.2	0.2±0.01
CEC (cmol/kg)	9±2	49±12	43±10
Clay (%)	52±5.5	-	-
MBI (meq/100g)	7.2±0.8	-	-
SFR	1.11±0.3	-	-
TC (%)	1.6±0.4	15.7±3.6	8.6±1.4
TOC (%)	1.2±0.06	13.6±3.5	7.2±1.1
TN (%)	0.05±0.01	0.35±0.1	0.3±0.04

EC – Electrical conductivity, SAR – Sodium absorption ratio, CEC – Cation exchange capacity, MBI – Methylene blue index, SFR – Sand to fine ratio, TC – Total carbon, TOC – Total organic carbon, TN – Total nitrogen, PMM – Peat mineral mix, Till – mineral substrate (classified as sandy clay loam soil with low organic carbon).

Table S3. Greenhouse temperature for the centrifuge (CF), co-mix (CM) and thickened tailings (TT) greenhouse bays from April to November 2019.

	TT Greenhouse Bay			CF/CM Tailings Greenhouse Bay		
Month	Temperature (°C)			Temperature (°C)		
	Min	Max	Average	Min	Max	Average
April	3.0	26.1	16.0	3.2	25.4	15.6
May	14.9	27.0	20.5	14.8	27.9	20.2
June	9.4	28.7	19.7	8.4	25.5	18.8
July	12.4	28.8	20.6	12.2	25.7	19.8
August	12.2	28.0	20.4	9.2	25.8	19.0
September	9.8	26.0	17.2	7.8	24.6	15.3
October	3.6	19.5	10.3	2.1	18.5	8.2
November	3.0	13.9	6.4	2.1	16.1	4.7

Table S4. Primer characteristics of genes of interest (GOIs) validated by qPCR.

Gene ID	Gene Description	Transcript ID	Primer sequence	Direction	Start position	End position	Tm (°C)	Amplicon Size (bp)
GOI-1	putative Myb family transcription factor At1g14600	DKX38_015522	CCAAAGGGGGTGGATACTGAAAAGG	F	456	480	66.4	142
		DKX38_015522	CCACTCCTCCCAAAACTACCCA	R	577	598	65.1	
GOI-2	ethylene-responsive transcription factor ABR1	DKX38_002262	CAACCGATAGAGCAGCAGGTGG	F	130	151	65.8	163
		DKX38_002262	CCGAGACCTAAAGCCCCGTATG	R	272	293	66.2	
GOI-3	desiccation-related protein PCC13-62	DKX38_025635	AGCTGGCTACAAACTGAACCCTC	F	550	572	65.3	164
		DKX38_025635	ACGCCCAGGAGTGACGCAACAA	R	693	714	67.7	
GOI-4	cytochrome P450 81E8	DKX38_001658	GCCCGTTTGCTCTTCCAATCATAGG	F	101	125	65.9	132
		DKX38_001658	ACGACCACGGCAAGGTAGGAT	R	213	233	66.1	
GOI-5	photosystem II repair protein PSB27-H1, chloroplastic	DKX38_002195	GATGCAGTGGCTAACCTGAGAGAAAC	F	304	329	65.5	144
		DKX38_002195	TCGGTCCAAAGCTAATGTAATGCCC	R	424	448	65.2	
GOI-6	protein NRT1/ PTR FAMILY 4.6	DKX38_018607	CACACTTGCTGCCACCTTTGTCTTG	F	286	310	65.4	121
		DKX38_018607	CGGAGCTGGATGGGGAAAAATGC	R	385	407	66.1	
GOI-7	shikimate O-hydroxycinnamoyltransferase	DKX38_028832	TCGACAACTCATTCCGACTGTTGACACTA	F	467	495	65.3	116
		DKX38_028832	TGCCACCCAACTCCGAGACAAAC	R	561	583	66.4	
GOI-8	xanthohumol 4-O-methyltransferase-like isoform X2	DKX38_020221	AGATGGAGGAGAAACACTCTTTGGGC	F	258	283	66.4	138
		DKX38_020221	ACATTGGCTCAAGCGATGACACGATTC	R	370	396	65.4	

Table S5. Primer characteristics of the reference housekeeping genes designed in this study.

Gene ID	Gene Description	Transcript ID	Primer sequence	Direction	Start position	End position	Tm (°C)	Amplicon Size (bp)
HKG-1	THO complex subunit 1	DKX38_000222	ATTTGGAGGAAGAGGCAGCAGC	F	1124	1145	65.3	101
		DKX38_000222	CAAGAACATGGCGGCGAAAACTTGG	R	1201	1225	65.2	
HKG-2	AP-4 complex subunit mu isoform X1	DKX38_002553	GTGGTCGATGCTGCACGTTTGC	F	787	808	65.3	112
		DKX38_002553	CCTGGCTCATTCGCTACAACAGAC	R	876	899	65.4	
HKG-3	AP-2 complex subunit mu	DKX38_004265	GTGGGGCTTTTGATGAAGATGCCA	F	592	615	64.8	140
		DKX38_004265	AGCGGACTCCTTCTTGTGTGATGTAA	R	707	732	64.8	
HKG-4	cleavage stimulation factor subunit 77 isoform X1	DKX38_004388	GGCTCAAACTGCACAGGAAGAGTC	F	878	91	65.4	173
		DKX38_004388	TTTGGTTGATATTCAGATATGAGTCCTTTGGC	R	1020	1051	64.2	
HKG-5	vacuolar protein sorting-associated protein 45 homolog	DKX38_008188	ACTGGAAAATTACCCAAAGACCAGCAG	F	913	939	64.9	120
		DKX38_008188	CCACCATCCTCTTGATGCTCATTCC	R	1009	1033	65.2	
HKG-6	26S proteasome regulatory subunit RPN13 isoform X2	DKX38_009318	GGGCTTGCTTCATTTTCAGTGGCTTG	F	250	275	66.2	199
		DKX38_009318	GTGAGTCCTCTTCAGCTTTTGGCTC	R	425	449	65.5	
HKG-7	serine/threonine-protein kinase TOUSLED isoform X2	DKX38_014729	GCTCGTATGGCTGGCAAGGTA	F	88	108	65.9	114
		DKX38_014729	TCGGACCCGAAGAAACAGAAGACC	R	179	202	65.5	
HKG-8	vacuolar protein sorting-associated protein 18 homolog isoform X1	DKX38_020249	ATCCTGGTGGTAGCCACTGTATCG	F	379	402	67.1	108
		DKX38_020249	CCTTTCAACCTCCCAAGCACACG	R	465	487	65.5	
HKG-9	THO complex subunit 5B	DKX38_020534	CGTCAGCAAGCGAATAAGGACAGTG	F	907	933	64.7	133
		DKX38_020534	GCTTCTTTGCTTTGAACCCTCTTTGGC	R	1014	1040	66.1	

Table S6. Read Summary of each RNA sequencing library

Group	Treatment	Replicate	Total reads	Reads mapped	Reads not mapped	Reads mapped (%)	Reads not mapped (%)
CF (Graminoid)	Control/Capping	CF1	31,973,396	28,541,117	3,432,279	89.27	10.73
		CF2	20,053,682	17,818,326	2,235,356	88.85	11.15
		CF3	22,572,200	19,565,026	3,007,174	86.68	13.32
		CF4	20,661,796	19,201,393	1,460,403	92.93	7.07
	Treatment/Tailings	CF5	2,357,121	2,155,456	201,665	91.44	8.56
		CF6	4,039,439	3,515,646	523,793	87.03	12.97
		CF7	23,895,315	21,617,688	2,277,627	90.47	9.53
	Average		17,936,136	16,059,236	1,876,900	89.52428571	10.47571429
CF (Shrubby)	Control/Capping	CF9	19,921,637	18,064,379	1,857,258	90.68	9.32
		CF10	10,300,594	8,731,923	1,568,671	84.77	15.23
		CF11	28,711,772	26,978,291	1,733,481	93.96	6.04
	Treatment/Tailings	CF14	37,407,726	25,997,957	11,409,769	69.5	30.5
		CF15	28,338,078	26,812,123	1,525,955	94.62	5.38
		CF16	27,423,888	26,299,568	1,124,320	95.9	4.1
	Average		25,350,616	22,147,374	3,203,242	88.23833333	11.76166667
CM (Group 1)	Control/Capping	CM2	6,879,000	6,461,828	417,172	93.94	6.06
		CM3	8,130,014	7,275,743	854,271	89.49	10.51
		CM4	8,167,200	7,683,717	483,483	94.08	5.92
	Treatment/Tailings	CM9	15,413,106	13,532,895	1,880,211	87.8	12.2
		CM11	10,716,719	9,588,814	1,127,905	89.48	10.52
		CM12	34,231,879	29,343,106	4,888,773	85.72	14.28
	Average		13,922,986	12,314,351	1,608,636	90	10
CM (Group 2)	Control/Capping	CM5	47,093,796	44,906,277	2,187,519	95.35	4.65
		CM6	38,868,637	36,745,161	2,123,476	94.54	5.46
		CM7	15,017,376	13,107,504	1,909,872	87.28	12.72
		CM8	14,772,366	10,424,878	4,347,488	70.57	29.43
	Treatment/Tailings	CM9	15,413,106	13,532,895	1,880,211	87.8	12.2
		CM11	10,716,719	9,588,814	1,127,905	89.48	10.52
		CM12	34,231,879	29,343,106	4,888,773	85.72	14.28

	Average		25,159,126	22,521,234	2,637,892	87.24857143	12.75142857
TT (Wetland)	Control/Capping	TT10	26,209,987	23,658,164	2,551,823	90.26	9.74
		TT11	27,798,599	25,857,806	1,940,793	93.02	6.98
		TT12	21,203,405	19,323,817	1,879,588	91.14	8.86
	Treatment/Tailings	TT13	24,918,368	22,980,412	1,937,956	92.22	7.78
		TT14	17,054,878	14,909,506	2,145,372	87.42	12.58
		TT15	14,235,219	11,949,487	2,285,732	83.94	16.06
		TT16	24,057,970	21,710,171	2,347,799	90.24	9.76
	Average		22,211,204	20,055,623	2,155,580	89.74857143	10.25142857
Grand average			20,916,013	18,619,563	2,296,450	89	11
